# Enhanced production of iturin A by strengthening fatty acid synthesis modules in *Bacillus amyloliquefaciens*

**DOI:** 10.3389/fbioe.2022.974460

**Published:** 2022-09-09

**Authors:** Lin Gao, Menglin She, Jiao Shi, Dongbo Cai, Dong Wang, Min Xiong, Guoming Shen, Jiaming Gao, Min Zhang, Zhifan Yang, Shouwen Chen

**Affiliations:** ^1^ State Key Laboratory of Biocatalysis and Enzyme Engineering, Environmental Microbial Technology Center of Hubei Province, College of Life Sciences, Hubei University, Wuhan, China; ^2^ Tobacco Research Institute, Chinese Academy of Agricultural Sciences, Qingdao, China; ^3^ Hubei Corporation of China National Tobacco Corporation, Wuhan, China; ^4^ Key Laboratory of Green Chemical Technology of Fujian Province University, College of Ecological and Resource Engineering, Wuyi University, Wuyishan, China

**Keywords:** iturin A, fatty acid, *Bacillus amyloliquefaciens*, metabolic engineering, gene expression

## Abstract

Iturin A is a biosurfactant with various applications, and its low synthesis capability limits its production and application development. Fatty acids play a critical role in cellular metabolism and target product syntheses, and the relationship between fatty acid supplies and iturin A synthesis is unclear. In this study, we attempted to increase iturin A production *via* strengthening fatty acid synthesis pathways in *Bacillus amyloliquefaciens*. First, acetyl-CoA carboxylase AccAD and ACP S-malonyltransferase *fabD* were overexpressed *via* promoter replacement, and iturin A yield was increased to 1.36 g/L by 2.78-fold in the resultant strain HZ-ADF1. Then, soluble acyl-ACP thioesterase derived from *Escherichia coli* showed the best performance for iturin A synthesis, as compared to those derived from *B. amyloliquefaciens* and *Corynebacterium glutamicum*, the introduction of which in HZ-ADF1 further led to a 57.35% increase of iturin A yield, reaching 2.14 g/L. Finally, long-chain fatty acid-CoA ligase LcfA was overexpressed in HZ-ADFT to attain the final strain HZ-ADFTL2, and iturin A yield reached 2.96 g/L, increasing by 6.59-fold, and the contents of fatty acids were enhanced significantly in HZ-ADFTL2, as compared to the original strain HZ-12. Taken together, our results implied that strengthening fatty acid supplies was an efficient approach for iturin A production, and this research provided a promising strain for industrial production of iturin A.

## Introduction

Lipopeptide biosurfactants are important secondary metabolites synthesized by microorganisms, which have important applications in petrochemical, agricultural, and medicinal fields. According to the structural components of the amino acid chain and fatty acids, lipopeptides are divided into iturin, surfactin, and fengycin families (Ongena and Jacques, 2008; Zhao et al., 2017). Among them, iturin A has the strongest antifungal activity in the iturin family, which is composed of seven amino acid residues Asn-Tyr-Asn-Gln-Pro-Asn-Ser and one β-amino fatty acid side chain (Chain length 14–17) (Tsuge et al., 2001). Due to the safety and environmental friendliness of biological control, iturin A has a good application prospect in the biological control of plant diseases (Xu et al., 2020). However, compared to the common lipopeptide surfactin, low-level synthesis capability limited iturin A application.

With the development of modern biotechnology, metabolic engineering and synthetic biology have been applied to the efficient synthesis of a variety of target products, thereby having an important impact in the fields of energy, chemical industry, medicine, and agriculture (Wang and Qi, 2009). Meanwhile, several attempts have been conducted to improve lipopeptide production. Through the introduction of 4-phosphopantetheinyl transferase Sfp, genome reduction, strengthening precursor (precursor amino acids and fatty acids) supplies, and surfactin synthetase cluster expression, surfactin produced by *Bacillus subtilis* reached 12.8 g/L (Wu et al., 2019). yerP was proven as the major surfactin exporter, and overexpression of yerP led to a 145% increase in surfactin production in *B. subtilis* (Li et al., 2015). Through simultaneous overexpression of regulators ComA and SigA, the iturin A yield was increased to 215 mg/L by 43-fold in *B. subtilis* ZK0 (Zhang et al., 2017). Meanwhile, genome shuffling was applied for rapid improvement of iturin A production strain, and iturin A was increased by 2.03-fold, as compared to the wild strain (Shi et al., 2018). In addition, promoter replacement has been proven as an efficient approach for lipopeptide production, which significantly increased surfactin (Jiao et al., 2017), iturin A (Xu et al., 2020), lichenysin (Qiu et al., 2014), and fengycin (Yaseen et al., 2016) yields. As for iturin A, few articles about genetic engineering breeding have been reported at present.

Fatty acids are important constituents of cell membrane phospholipids, which also play a key role in the syntheses of target products (Wang M. et al., 2019), and free fatty acids and their derivatives are used in the fields of industrial chemicals, pharmaceuticals, agriculture, etc. (Bentley et al., 2016). Previously, acyl-ACP thioesterase, propionyl-CoA synthase, and β-ketoacyl-acyl carrier protein synthase III were engineered, which significantly increased odd straight-chain free fatty acid production (Wu and San, 2014). A metabolic switch was applied to dynamically regulate fatty acid biosynthesis in *Escherichia coli* and led to a 2.1-fold increase in the fatty acid titer (Xu et al., 2014). In addition, the fatty acid supply served a critical role in lipopeptide synthesis as a fatty acid chain is contained in the structure of lipopeptide. Through deleting the regulator gene *codY* and 2-oxoisovalerate dehydrogenase gene *lpdV*, the yield of the surfactin C14 isoform was significantly enhanced (Dhali et al., 2017). Strengthening branched-chain fatty acid biosynthetic pathways was proven as an efficient approach for surfactin production, which led to a 1.2-fold increase in surfactin production (Wu et al., 2019). In addition, the antisense RNA strategy was applied to identify the critical role of the biotin carboxylase II gene *yngH* in malonyl-CoA and surfactin syntheses, and overexpression of *yngH* led to a 43% increase of surfactin production in *B. subtilis*, reaching 13.37 g/L (Wang M. et al., 2019). However, the relationship between fatty acid synthesis and iturin A production has not been studied so far.

**FIGURE 1:**
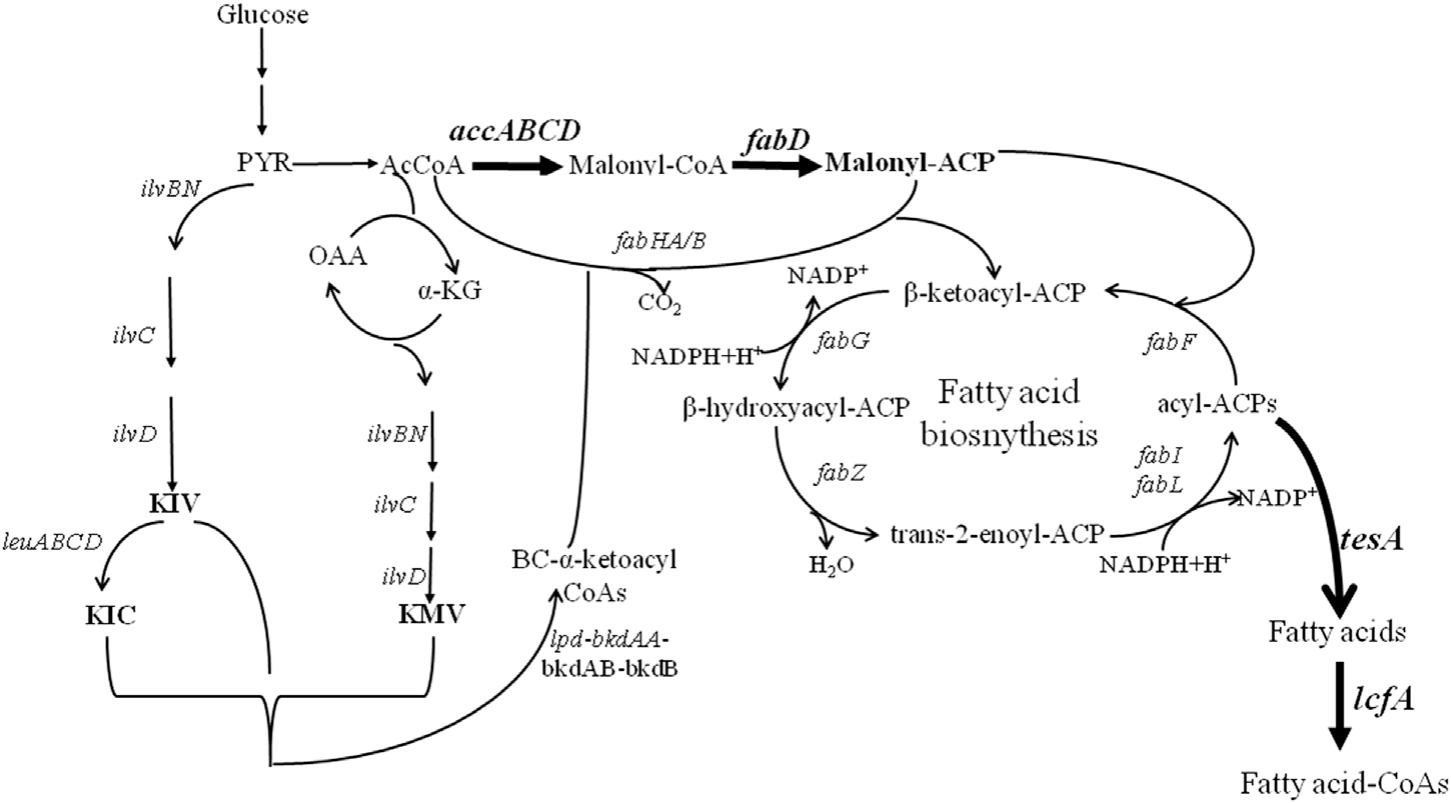
Metabolic engineering of fatty acid synthesis pathways for enhanced production of iturin A.

The low synthesis capability of iturin A limits its application promotion, although several approaches of genetic engineering breeding and fermentation process optimization have been developed to improve iturin A production (Mizumoto et al., 2007; Mizumoto and Shoda, 2007; Jin et al., 2015; Zhang et al., 2017; Shi et al., 2018; Dang et al., 2019; Xu et al., 2020; Wang et al., 2021). To improve iturin A synthesis, fatty acid biosynthetic pathways were systematically strengthened in *Bacillus amyloliquefaciens* HZ-12, a wide-type strain with the synthesis capability of iturin A (Xu et al., 2020), and the synthesis modules of malonyl-CoA, malonyl-ACP, free fatty acid, and fatty acid-CoA were strengthened for iturin A production (Figure 1). Our results indicated that strengthening fatty acid supplies was an efficient approach for iturin A production, whose strategy could also be applied in the production of other metabolites.

## Materials and methods

Strains, plasmids, and cultivation conditions: *B. amyloliquefaciens* HZ-12 acted as the original strain for recombinant strain construction. The plasmids pHY300PLK and T_2_(2)-Ori were applied for gene overexpression, promoter replacement, and gene integration, respectively. The primers used in this research are provided in Table 1 and Supplementary Table S1 (Refer to the Supplementary information). *E. coli* DH5α acted as the host for plasmid construction. *B. amyloliquefaciens* and *E. coli* were cultivated at 28°C and 37°C, respectively, and a corresponding antibiotic (20 mg/L kanamycin or tetracycline) was added when necessary. For iturin A production, strains were cultivated in 250-ml flasks containing 50 ml of LB medium for 12 h, then transferred (1 ml) into a 250-ml flask containing 25 ml iturin A production medium (g/L, 30 corn starch, 70 soybean meal, 1.0 K_2_HPO_4_·3H_2_O, 1.0 MgSO_4_·7H_2_O, 1.0 FeSO_4_·7H_2_O, 0.01 MnSO_4_·H_2_O, and natural pH), and further cultivated at 28°C, 230 rpm for 72 h. Since, the iturin A fermentation medium is not conducive to bacterial isolation and fatty acid extraction, medium E (ME medium, g/L, 20 glucose, 20 sodium glutamate, 10 sodium citrate, 7 NH_4_Cl, 0.5 K_2_HPO_4_·3H_2_O, 0.5 MgSO_4_·7H_2_O, 0.04 FeCl_3_·6H_2_O, 0.104 MnSO_4_·H_2_O, and 0.15 CaCl_2_·2H_2_O) was applied for fatty acid determination, and the mid-logarithmic fermentation (20 h) samples were attained for the determination of fatty acid contents.

**TABLE 1:** Strains and plasmids used in this research.

Strain or plasmid	Relevant genotype/description	Source
Strain		
*B. subtilis* 168	Wild type	Lab collection
*B. licheniformis* DW2	CCTCC M2011344	Lab collection
*B. amyloliquefaciens*		
HZ-12	Iturin A production strain and wild-type (CCTCC M2015234)	Lab collection
HZ/pHY300	Derivative of HZ-12, harboring the plasmid pHY300 as the control strain	This work
HZ/pHY-AccAD	Derivative of HZ-12, harboring the AccAD expression plasmid pHY-AccAD	This work
HZ/pHY-AccBC	Derivative of HZ-12, harboring the AccBC expression plasmid pHY-AccBC	This work
HZ-AD1	Derivative of HZ-12 and the promoter of genes *accAD* was replaced by P43	This work
HZ-AD2	Derivative of HZ-12 and the promoter of genes *accAD* was replaced by P_bacA_	This work
HZ-AD3	Derivative of HZ-12 and the promoter of genes *accAD* was replaced by P_dual3_	This work
HZ-AD2/pHY300	Derivative of HZ-AD2, harboring the plasmid pHY300 as the control strain	This work
HZ-AD2/pHY-*fabD*	Derivative of HZ-AD2, harboring the *fabD* expression plasmid pHY-*fabD*	This study
HZ-ADF1	Derivative of HZ-AD2 and the promoter of gene *fabD* was replaced by P43	This study
HZ-ADF2	Derivative of HZ-AD2 and the promoter of gene *fabD* was replaced by P_bacA_	This study
HZ-ADF3	Derivative of HZ-AD2 and the promoter of gene *fabD* was replaced by P_dual3_	This study
HZ-ADF1/pHY300	Derivative of HZ-ADF1, harboring the plasmid pHY300 as the control strain	This study
HZ-ADF1/pHY-TesA_Ba_	Derivative of HZ-ADF1, harboring the TesA expression plasmid pHY-TesA_Ba_	This study
HZ-ADF1/pHY-TesA_Ec_	Derivative of HZ-ADF1, harboring the TesA expression plasmid pHY-TesA_Ec_	This study
HZ-ADF1/pHY-TesA_Cg_	Derivative of HZ-ADF1, harboring the TesA expression plasmid pHY-TesA_Cg_	This study
HZ-ADFT	Derivative of HZ-ADF1 and TesA integration expression strain	This study
HZ-ADFT/pHY300	Derivative of HZ-ADFT, harboring the plasmid pHY300 as the control strain	This study
HZ-ADFT/pHY-LcfA	Derivative of HZ-ADFT, harboring the LcfA expression plasmid pHY-LcfA	This study
HZ-ADFTL1	Derivative of HZ-ADFT and the promoter of gene *lcfA* was replaced by P43	This study
HZ-ADFTL2	Derivative of HZ-ADFT and the promoter of gene *lcfA* was replaced by P_bacA_	This study
HZ-ADFTL3	Derivative of HZ-ADFT and the promoter of gene *lcfA* was replaced by P_dual3_	This study
Plasmid		
T_2_ (2)-Ori	*E. coli*−*Bacillus* shuttle vector; Ori_pUC_/Ori_ts_, and Kan^r^	Lab collection
T2-P43-AccAD	Promoter P43 with the upstream and downstream homogenous arms of P_accAD_ inserted into T_2_ (2)-ori	This work
T2-PbacA-AccAD	Promoter P_bacA_ with the upstream and downstream homogenous arms of P_accAD_ inserted into T_2_ (2)-ori	This work
T2-Pdual3-AccAD	Promoter P_dual3_ with the upstream and downstream homogenous arms of P_accAD_ inserted into T_2_ (2)-ori	This work
T2-P43-*fabD*	Promoter P43 with the upstream and downstream homogenous arms of P_fabD_ inserted into T_2_ (2)-ori	This study
T2-PbacA-*fabD*	Promoter P_bacA_ with the upstream and downstream homogenous arms of P_fabD_ inserted into T_2_ (2)-ori	This study
T2-Pdual3-*fabD*	Promoter P_dual3_ with the upstream and downstream homogenous arms of P_fabD_ inserted into T_2_ (2)-ori	This study
T2-TesA	TesA integration expression vector	This study
T2-P43-LcfA	Promoter P43 with the upstream and downstream homogenous arms of P_lcfA_ inserted into T_2_ (2)-ori	This study
T2-PbacA-LcfA	Promoter PbacA with the upstream and downstream homogenous arms of P_lcfA_ inserted into T_2_ (2)-ori	This study
T2-Pdual3-LcfA	Promoter Pdual3 with the upstream and downstream homogenous arms of P_lcfA_ inserted into T_2_ (2)-ori	This study
pHY300PLK	*E. coli* and *B s* shuttle vector; Amp^r^ and Tet^r^	Lab collection
pHY-AccAD	AccAD expression vector based on pHY300PLK	This study
pHY-AccBC	AccBC expression vector based on pHY300PLK	This study
pHY-*fabD*	*fabD* expression vector based on pHY300PLK	This study
pHY-TesA_Ba_	TesA_Ba_ expression vector based on pHY300PLK	This study
pHY-TesA_Ec_	TesA_Ec_ expression vector based on pHY300PLK	This study
pHY-TesA_Cg_	TesA_Cg_ expression vector based on pHY300PLK	This study
pHY-LcfA	LcfA expression vector based on pHY300PLK	This study

Construction of gene overexpression strains: gene overexpression strain was constructed according to our previously reported method (Cai et al., 2018), and the construction procedure of genes *accAD* overexpression strain served as an example. In brief, the P43 promoter from *B. subtilis* 168, genes *accAD* from *B. amyloliquefaciens* HZ-12, and *amyL* terminator from *B. licheniformis* DW2 were amplified by corresponding primers (Supplementary Table S1), and these three fragments were fused through splicing by Overlap Extension (SOE)-PCR. The fused fragment was then inserted into the plasmid pHY300PLK at restriction enzyme sites *Eco*RI/*Xba*I, and diagnostic PCR and DNA sequences were used to confirm that the gene expression plasmid pHY-AccAD was constructed successfully. The plasmid pHY-AccAD was electro-transferred into *B. amyloliquefaciens* HZ-12 to attain the gene overexpression strain HZ/pHY-AccAD. Similarly, other genes’ (*accBC*, *fabD*, *tesA,* and *lcfA*) overexpression strains were constructed by the same method.

Construction of the promoter replacement strain: promoter replacement was proven as an efficient approach for gene expression regulation, and the promoter replacement strain was constructed based on our previously reported research (Xu et al., 2020), and the construction procedure of the gene *accAD* promoter replacement strain served as an example. Briefly, the upstream and downstream homologous arms of the gene *accAD* promoter from *B. amyloliquefaciens* HZ-12 and the P43 promoter from *B. subtilis* 168 were amplified by corresponding primers and fused through SOE-PCR. The fused fragment was inserted into plasmid T_2_ (2)-Ori at restriction enzyme sites *Sac*I/*Xba*I, and diagnostic PCR and DNA sequences were used to confirm that the promoter replacement plasmid T2-P43-AccAD was constructed successfully.

Subsequently, the plasmid T2-P43-AccAD was electro-transferred into *B. amyloliquefaciens*, and transformants were verified by colony PCR. The positive transformant was cultivated in an LB medium with 20 mg/L kanamycin at 45°C for three generations and then transferred into the kanamycin-free medium at 37°C for six generations. The kanamycin-sensitive colonies were verified by diagnostic PCR to attain double-cross transformants and further confirmed by DNA sequencing. Similarly, other genes’ (*fabD* and *lcfA*) promoter replacement strains were constructed by the same method.

Construction of gene integration expression strain: integration expression is an important strategy to achieve stable expression of the heterologous gene. Here, the acyl-ACP thioesterase TesA integration expression strain was attained according to our previously reported research (Cai et al., 2018). Briefly, the upstream and downstream homologous arms, and the gene *tesA* expression cassette were amplified and fused through SOE-PCR. Then, the fused fragment was inserted into T_2_ (2)-Ori to construct the *tesA* integration expression vector T2-TesA. Then, T2-TesA was electro-transferred into *B. amyloliquefaciens*, and the *tesA* integration expression strain was attained by homologous double exchanges, similar to the construction process of promoter replacement, verified by diagnostic PCR and DNA sequences.

Determination of gene transcriptional levels: total RNA was extracted by TRIzol^®^ reagent, the trace DNA was digested by DNase I, and the first strand of cDNA was amplified using the RevertAid First Strand cDNA Synthesis Kit (Thermo, United States). The *16S rRNA* from *B. amyloliquefaciens* HZ-12 was used as the reference gene. The gene transcriptional levels of recombinant strains were compared with those of the control strain after being normalized to the reference gene *16S rRNA*. All the experiments were performed in triplicates.

Analytical methods: the cell biomass was measured by a dilution coating method, and iturin A yields of various strains were determined by high-performance liquid chromatography (HPLC) using a 1260 HPLC system (Agilent Technologies, USA) equipped with the Agilent LiChrospher C18 column (4.6 mm × 250 mm, 5 μm), according to our previous research (Xu et al., 2020). The mobile phase was 10 mmol/L ammonium acetate/acetonitrile = 65:35 (V/V), and the flow rate was 1.0 ml/min. The injection volume was 10 μl, the detection wavelength was 210 nm, and the concentration of iturin A was calculated by the standard curve made by standard iturin A (Sigma, CAS 52229-90-0, purity 95%). The concentrations of total reducing sugar were determined by the DNS (3, 5-dinitrosalicylic acid) method (Hsiao et al., 2019), according to the standard curve line made by glucose.

To determine the contents of fatty acids, 2 ml of the fermentation broth was centrifuged, and the cell pellet was washed with normal saline. Then, the cell pellet was dissolved in 1 ml of solution I (15% NaOH, 50% methanol) and insulated at 100°C for 5 min. The volume of 2 ml of solution II (3 mol/L HCL, 36% methanol) was added into the mixture and insulated at 80°C for 10 min. The volume of 1.25 ml of n-hexane was added and mixed. After aspirating the upper organic phase, 3 ml of the 1.2% NaOH mixture and 300 μl of the saturated sodium chloride solution were added, and the upper liquid phase was attained for fatty acid analysis, conducted using a gas chromatography–mass spectrometry (GC-MS) system equipped with the TG-5MS column (30 m × 0.25 mm, Thermo). The carrier gas helium had a constant flow rate of 0.8 ml/min, the splitless injection mode was used, and the temperature of the injection port was 250°C. The initial temperature of the column oven was 40°C and maintained for 5 min, and the temperature was raised to 150°C with the rate of 50°C/min and maintained for 2 min. The final ramping was carried out at 240°C in 1 min and then kept for 10 min. The MS transfer line temperature and ion source temperature were 280°C. The scan masses (m/z) ranged from 50 to 700. The concentrations of fatty acids were calculated based on the standard cure made by fatty acid methyl ester standards, and phenethyl acetate was severed as the internal standard for fatty acid detection (Tan et al., 2016; Liu et al., 2017).

As for the determination of free fatty acids, cells were disrupted by ultrasound and centrifuged, and the supernatant was derivatized for free fatty acid analysis, according to the aforementioned method.

Statistical analyses: all samples were analyzed in triplicate, and all data were conducted to analyze the variance at *p* < 0.05 and *p* < 0.01, and a *t*-test was applied to compare the mean values using the software package Statistica 6.0 (Cai et al., 2018).

## Results

Strengthening acetyl-CoA carboxylase for iturin A production: previously, acetyl-CoA carboxylase, encoding four genes *accABCD*, was proven as the rate-limiting step for malonyl-CoA and fatty acid syntheses (Wu and San, 2014), and strengthening acetyl-CoA carboxylase expression benefited surfactin production (Wang M. et al., 2019). Here, to enhance acetyl-CoA carboxylase expression for iturin A production, genes *accAC* and *accBD* overexpression vectors were constructed and electro-transferred into *B. amyloliquefaciens* HZ-12 to attain recombinant strains HZ/pHY-AccAD and HZ/pHY-AccBC, respectively. Meanwhile, the plasmid pHY300PLK was transferred to *B. amyloliquefaciens* HZ-12 to attain HZ/pHY300 as the control strain.

**FIGURE 2:**
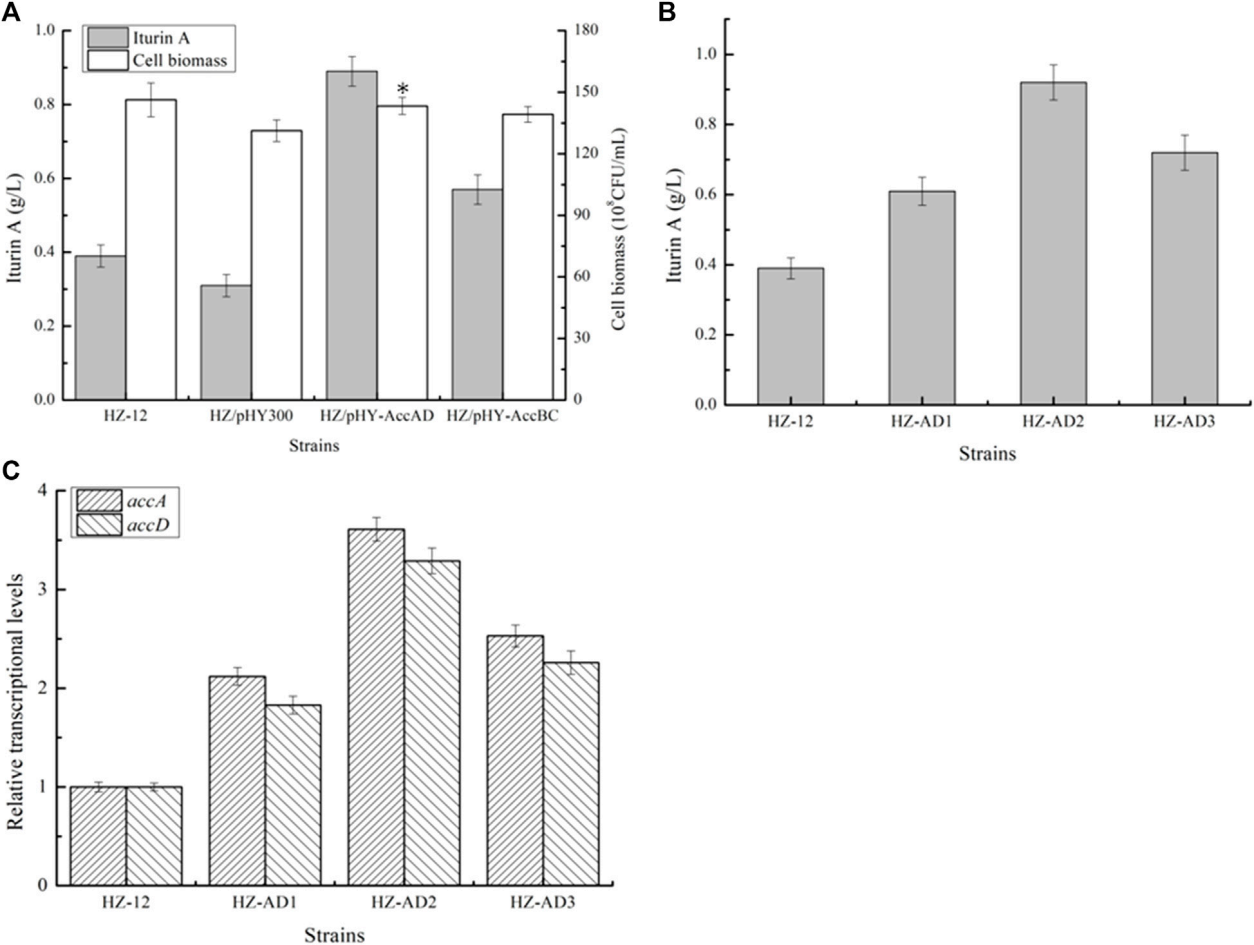
Effect of acetyl-CoA carboxylase overexpression on iturin A production. **(A)** Effects of strengthening AccAD and AccBC expressions on iturin A production. **(B)** Iturin A yields of AccAD overexpression strains mediated by different promoters. **(C)** Transcriptional level analysis.

Then, all these recombinant strains were cultivated in an iturin A production medium, and cell biomass and iturin A yields were measured at the end of fermentation. Based on our results, as shown in Figure 2A, strengthening *accAC* and *accBD* benefited cell growth, which was increased by 9.15% and 6.09%, respectively, as compared to HZ/pHY300, and iturin A yields of HZ/pHY-AccAD and HZ/pHY-AccBC were 0.89 g/L and 0.57 g/L, which increased by 1.87- and 0.84-fold, respectively. Therefore, our results indicated that strengthening acetyl-CoA carboxylase expression benefited iturin A synthesis.

Due to the influence of the free plasmid on the metabolic burden and subsequent genetic engineering (Zhou et al., 2017a), the promoter of the gene *accAD* was replaced with the well-recognized strong promoter P43 from *B. subtilis* 168, P_bacA_ from *B. licheniformis* DW2 (Rao et al., 2021), and dual-promoter P_dual3_ constructed in our previous research (Rao et al., 2020) to attain promoter replacement strains HZ-P43-AD (HZ-AD1), HZ-PbacA-AD (HZ-AD2), and HZ-Pdual3-AD (HZ-AD3), respectively. Based on our results, as shown in Figure 2, transcriptional levels of genes *accA* and *accD* were all increased in the recombinant strains, and HZ-AD2 showed the best performance on iturin A synthesis, which consisted of the results of transcriptional levels, and iturin A yield produced by HZ-AD2 reached 0.92 g/L (Figure 2), increasing 1.36-folds as compared to the control strain HZ-12.

**FIGURE 3:**
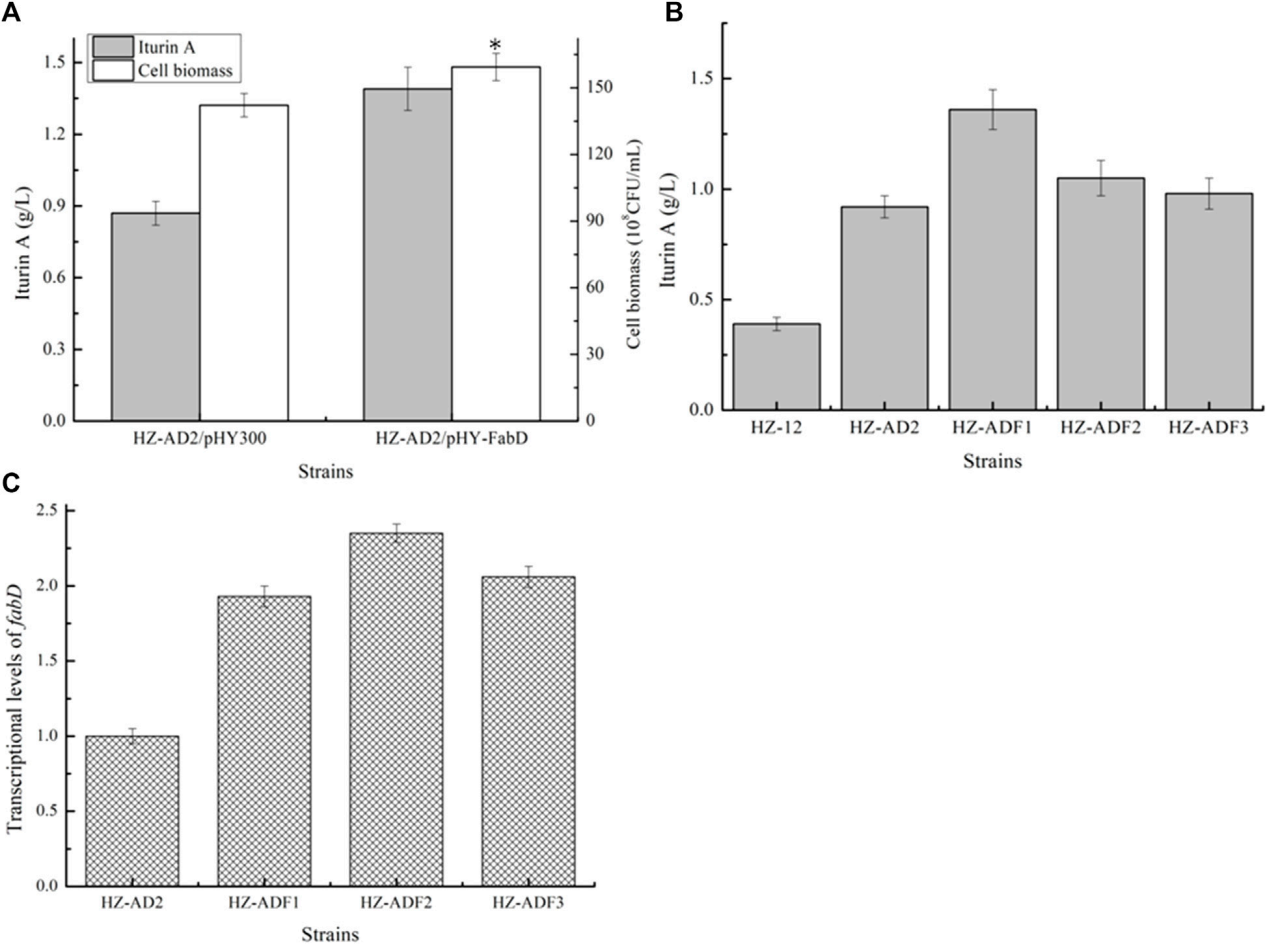
Effect of ACP S-malonyltransferase overexpression on iturin A production. **(A)** Effects of ACP S-malonyltransferase *fabD* overexpression on iturin A production. **(B)** Iturin A yields of *fabD* overexpression strains mediated by different promoters. **(C)** Transcriptional level analysis.

Overexpression of ACP S-malonyltransferase *fabD* for iturin A synthesis: ACP S-malonyltransferase *fabD* functions to catalyze malonyl-CoA to malonyl-ACP (Bentley et al., 2016), which provides carbon donors for fatty acid synthesis. Here, gene *fabD* overexpression strain HZ-AD2/pHY-*fabD* was attained based on the strain HZ-AD2, and iturin A yield, which was increased to 1.39 g/L by 59.77%, indicated that the insufficient *fabD* expression might be an important factor limiting iturin A synthesis. Meanwhile, the gene *fabD* promoter P_fabD_ was replaced with P43, P_bacA_, and P_dual3_, attaining recombinant strains HZ-AD2-P43-*fabD* (HZ-ADF1), HZ-AD2-PbacA-*fabD* (HZ-ADF2), and HZ-AD2-Pdual3-*fabD* (HZ-ADF3), respectively. Based on our results, the increasing trends of *fabD* transcriptional levels were consistent with those of *accAD*; however, the maximum iturin A yield was attained under the mediation of the promoter P43, although the *fabD* transcriptional level of HZ-ADF1 was lower than those of HZ-ADF2 and HZ-ADF3, and the iturin A yield of HZ-ADF1 reached 1.36 g/L, increasing by 47.83% as compared to the strain HZ-AD2 (0.89 g/L) (Figure 3).

**FIGURE 4:**
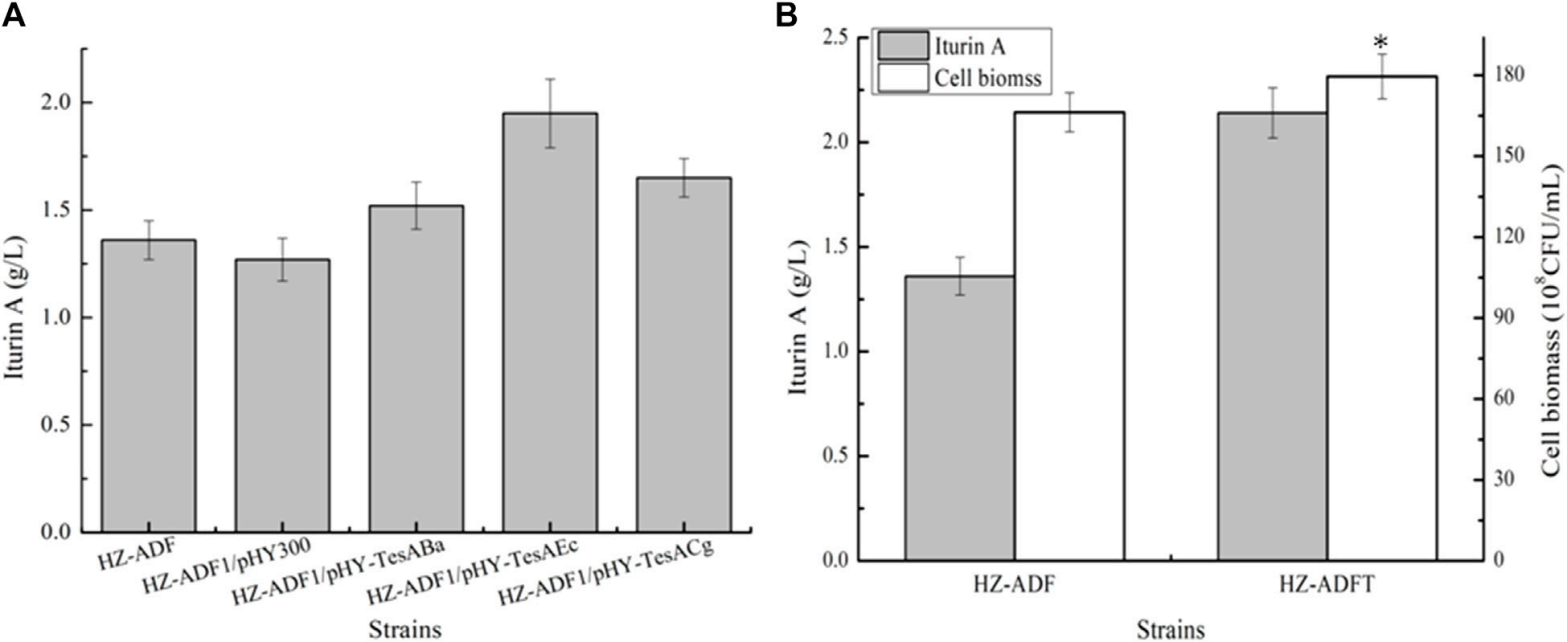
Strengthening soluble acyl-ACP thioesterase expression for iturin A production. **(A)** Effects of overexpression of acyl-ACP thioesterase from different species on iturin A production. **(B)** Introducing acyl-ACP thioesterase from *E. coli* for iturin A production.

Strengthening soluble acyl-ACP thioesterase TesA for iturin A production: thioesterase catalyzes fatty acid ACP to free fatty acid, which controls the fatty acid synthesis and chain length (Deng et al., 2020). Here, acyl-ACP thioesterase TesA from *B. amyloliquefaciens* HZ-12, *E. coli* DH5α, and *Corynebacterium glutamicum* 13,032 were overexpressed in HZ-ADF1 and attained recombinant strains HZ-ADF1/pHY-TesA_Ba_, HZ-ADF1/pHY-TesA_Ec_, and HZ-ADF1/pHY-TesA_Cg_, respectively. Furthermore, all these strains were cultivated in an iturin A production medium, and the results of Figure 4A indicated that the overexpression of acyl-ACP thioesterase benefited iturin A synthesis, whose yields were increased by 19.69%, 53.54%, and 29.92% in HZ-ADF1/pHY-TesA_Ba_, HZ-ADF1/pHY-TesA_Ec_, and HZ-ADF1/pHY-TesA_Cg_, respectively, suggesting that insufficient thioesterase expression restricted iturin A synthesis, and overexpression of acyl-ACP thioesterase from *E. coli* showed the best performance. Meanwhile, cell biomass was increased by 8.00% in acyl-ACP thioesterase overexpression strains. Furthermore, the gene *tesA* from *E. coli* was integrated and expressed in HZ-ADF1, mediated by the promoter P43, attaining the recombinant strain HZ-ADFT. As shown in Figure 3B, 1.95 g/L iturin A was produced by HZ-ADFT, increasing by 43.38% as compared to HZ-ADF1.

**FIGURE 5:**
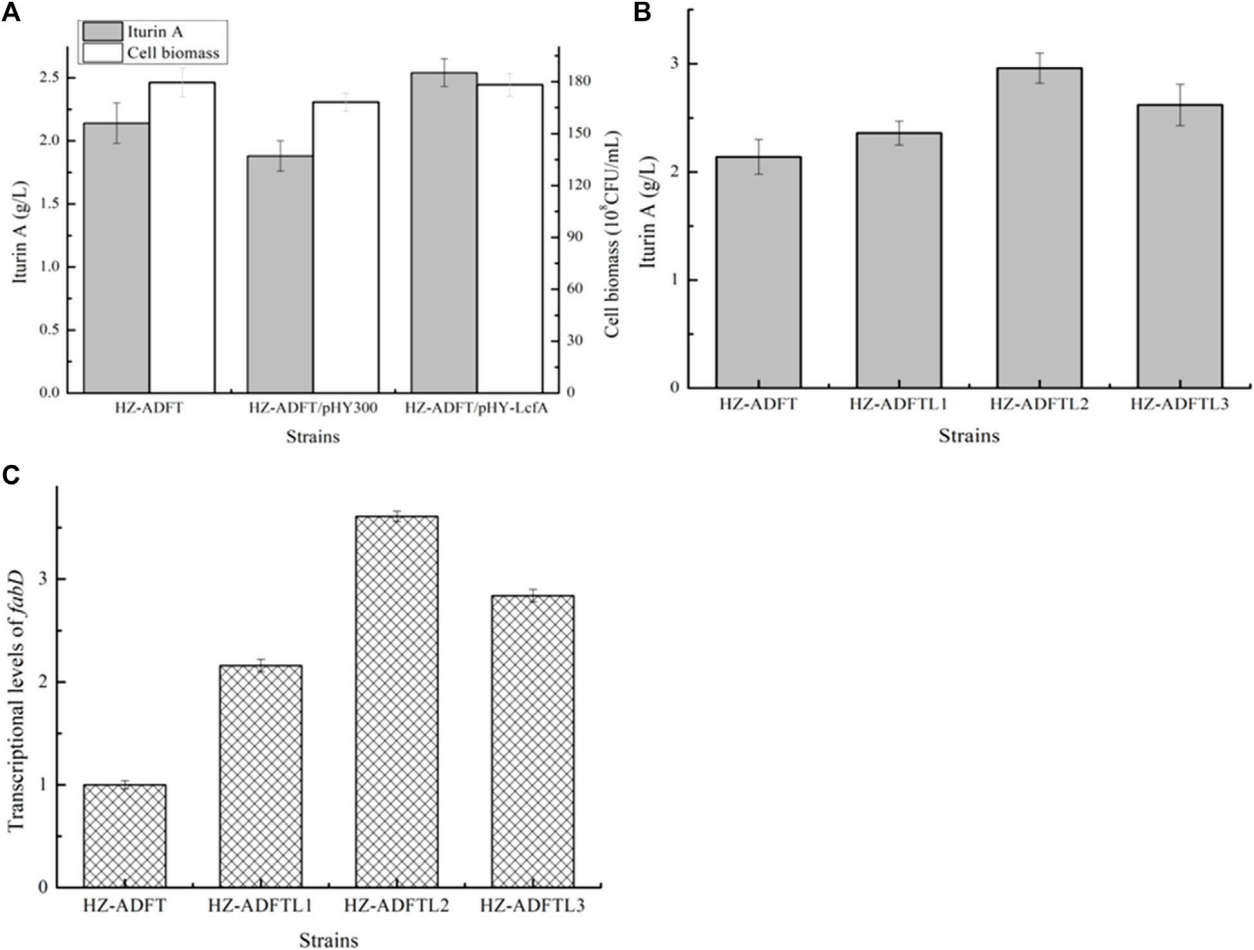
Effect of long-chain fatty acid-CoA ligase overexpression on iturin A production. **(A)** Effects of long-chain fatty acid-CoA ligase LcfA overexpression on iturin A production. **(B)** Iturin A yields of LcfA overexpression strains mediated by different promoters. **(C)** Transcriptional level analysis.

Effects of long-chain fatty acid-CoA ligase LcfA overexpression on iturin A production: fatty acids will convert to fatty acyl-CoAs for iturin A synthesis, under the catalysis of long-chain fatty acid synthetase (Bae et al., 2014). Here, long-chain fatty acid synthetase LcfA was overexpressed in HZ-ADFT and attained the strain HZ-ADFT/pHY-LcfA. Our results implied that LcfA overexpression led to a 35.11% increase in iturin A yield (Figure 5A), which also benefited cell growth. Furthermore, a promoter replacement approach was conducted to enhance *lcfA* expression, and our results implied that the gene *lcfA* mediated by promoter P_bacA_ showed the best performance, and iturin A yield of the resulting strain HZ-ADFTL2 was increased to 2.96 g/L by 38.32% as compared to the strain HZ-ADFT (Figure 5).

**FIGURE 6:**
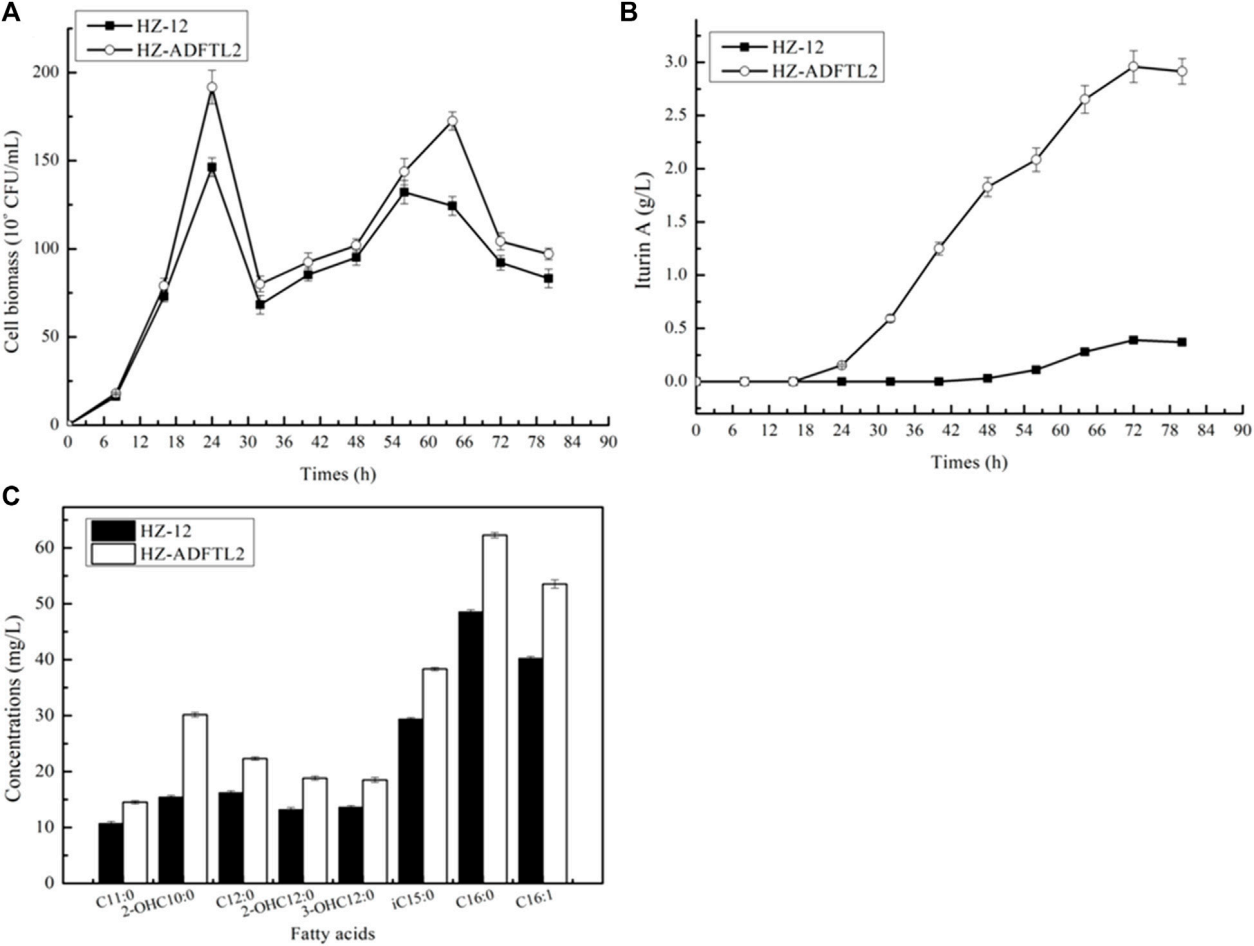
Fermentation analysis of *B. amyloliquefaciens* HZ-12 and HZ-ADFTL2 strains. **(A)** Cell biomass, **(B)** iturin A yields, and **(C)** fatty acid concentrations.

Fermentation analyses of *B. amyloliquefaciens* HZ-12 and HZ-ADFTL2: *B. amyloliquefaciens* HZ-12 and HZ-ADFTL2 were cultivated in an iturin A production medium, and cell biomasses and iturin A yields were determined throughout the fermentation process. Based on our results, as shown in Figure 6, HZ-ADFTL2 showed a shorter lag period, and the maximum cell biomass reached 191.75*10^8^ CFU/ml, increasing by 31.08% as compared to HZ-12 (146.29*10^8^ CFU/ml). Meanwhile, the biomasses of these two strains were decreased at 24 h, ascribing to cell autolysis. After that, cell biomasses continued to increase for 32 h due to secondary growth, and the cell was autolysed for 60 h, until carbon resource was consumed (Supplementary Figure S1).

In addition, iturin A was synthesized for 24 h at a stable period, and iturin A yields of HZ-ADFTL2 were higher than those of HZ-12 throughout the fermentation process, and the maximum yield reached 2.96 g/L, increasing by 6.59-fold compared to HZ-12 (0.39 g/L). To determine fatty acid concentrations, these strains were cultivated in the ME medium, and iturin A yields produced by HZ-ADFTL2 were also higher than those of HZ-12 in the ME medium (Supplementary Figure S2). Meanwhile, fatty acid contents of these two strains were measured at the mid-logarithmic phase in the ME medium, and fatty acid contents of HZ-ADFTL2 were higher than those of HZ-12 (Figure 6C). Additionally, free fatty acid concentrations were also determined, and several free fatty acids were detected by HZ-ADFTL2 (Supplementary Figure S3), while no free fatty acid was detected in the control strain HZ-12 due to the lack of thioesterase. Our results indicated that strengthening these aforementioned key pathways improved fatty acid supplies, which was conducive to cell growth and iturin A production.

## Discussion

Due to its features of having good surface activity and antifungal properties, iturin A has important application prospects in the areas of petrochemical, agricultural, and pharmaceutical industries (Ongena and Jacques, 2008). At present, there are few reports on the high-level production of iturin A, and low synthetic capability limits its production and application development. In this research, fatty acid supplies were strengthened by strengthening acetyl-CoA carboxylase, ACP S-malonyltransferase, acyl-ACP thioesterase, and long-chain fatty acid-CoA ligase; 2.96 g/L iturin A was produced by the final strain HZ-ADFTL2, which increased by 6.59-fold as compared to the original strain. Taken together, our results confirmed that strengthening fatty acid synthesis was an efficient approach for iturin A production, and this research provided a promising strain for industrial production of iturin A.

In recent years, with the continuous development of synthetic biology, various metabolic engineering breeding strategies have been developed for the efficient production of target products (Wang and Qi, 2009). As for lipopeptides, most research studies were focused on the synthetic regulation, metabolic engineering breeding, and surfactin application, and the strategies of the synthetase cluster overexpression, strengthening precursor (amino acids and fatty acids) supplies and lipopeptide exporters, rewiring regulators’ expression, etc., were also developed to improve surfactin production (Coutte et al., 2015; Li et al., 2015; Dhali et al., 2017; Hu et al., 2019; Wu et al., 2019). Compared with the antibacterial activity of surfactin, iturin A is mainly fungicidal, and the amino acid composition of iturin A is complex. Meanwhile, iturin A yield produced by *Bacillus* is relatively low, although several pieces of research on fermentation process optimization have been conducted to improve its yield (Jin et al., 2015; Wang et al., 2021). Previously, by replacing the promoter of the iturin A synthetase cluster and deleting the negative regulator gene *abrB* in *B. amyloliquefaciens*, iturin A yield was increased by 1.81-fold (Xu et al., 2020). Here, our research implied that strengthening fatty acid syntheses was confirmed as an efficient approach, and the iturin A yield was increased to 2.96 g/L in the strain HZ-ADFTL2. However, the iturin A yield attained in this research is still lower than the current yields of surfactin produced by *Bacillus* (more than 10 g/L) (Wang M. et al., 2019; Wu et al., 2019), indicating that more studies should be conducted to engineer strains for high-level production of iturin A.

Fatty acid synthesis is one of the indispensable basic metabolic pathways of all living organisms, and fatty acids are the main component of the cell membrane and play an important role in cell signal transduction and energy storage (Xu et al., 2014). The conversion of acetyl-CoA to malonyl-CoA, which is catalyzed by acetyl-CoA carboxylase, is the first and rate-limiting step of fatty acid synthesis (Wang M. et al., 2019). Satoh et al. (2020) have enhanced the expressions of *accABCD* and pantothenate kinase in *E. coli*, and the yield of fatty acid was increased 5.60-fold. Biotin carboxylase II *yngH* was proven to play a critical role in maintaining acetyl-CoA carboxylase, and overexpression of *yngH* led to a 43% increase in surfactin production (Wang M. et al., 2019). Fatty acids are important precursors of iturin A synthesized by *Bacillus*, and it was found that the enhanced expression of acetyl-CoA carboxylase could improve iturin A production. Compared with the initial strain, overexpression of the gene *accAD* increased iturin A yield by 135.90%. ACP S-malonyltransferase *fabD* catalyzes the second step in the first stage of fatty acid synthesis, which catalyzes the transfer of malonyl-CoA to the sulfhydryl group by the “Ping-Pong mechanism”. As *fabD* is the only enzyme that catalyzes the initial substrate onto ACP, it plays an irreplaceable role in fatty acid syntheses (Guo et al., 2014). In this study, enhanced expression of the gene *fabD* increased fatty acid supplies, and iturin A yield was further enhanced by 47.83%. Previous studies have shown that acetyl-CoA carboxylase is inhibited by acyl-ACP, and coexpression of ACP thioesterase with acetyl-CoA carboxylase will decouple the utilization of acyl-ACP from phospholipid synthesis (Tamano et al., 2013; Evans et al., 2017), and this is also the reason for the significant increase of iturin A yield in *tesA* overexpression strain. When the activity of fatty acid synthetase is high, malonyl-CoA will continue to be catalyzed into fatty acids. We continued to superposition and strengthen the expression of long-chain fatty acid-CoA ligase LcfA, which significantly increased iturin A production. In addition, *Bacillus* was proven as a wonderful strain for acetoin and 2, 3-butanediol production due to excess accumulation of pyruvate (Fu et al., 2016; Qiu et al., 2016). Here, more acetyl-CoA was used for fatty acid synthesis, which directed the carbon metabolic flux from pyruvate to acetyl-CoA without affecting the TCA cycle, and cell biomass of HZ-ADFTL2 was higher than that of the control strain.

With the deepening of the understanding of synthetic biology and metabolic pathways, people have found that a single intensity of gene expression regulation often cannot achieve optimal breeding effects (Rao et al., 2021). Because of this, several expression element libraries have been developed to optimally regulate the expression levels of target genes (Zhou et al., 2017b). Previously, a gradient promoter library was obtained *via* manipulating the core spacer of the promoter P_srfA_ of *B. subtilis* 168, which has been applied in the high-level production of target protein production (Wang Y. et al., 2019). Our group has also established a promoter library *via* manipulating 5′-UTR of the promoter P_bacA_, and the attained promoter library was applied in the expression regulation of heterologous proteins, bacitracin synthetase cluster, and metabolic pathways (Rao et al., 2021). Here, three promoters with different intensities were applied for the optimization of synthesis pathways of fatty acids, and our results indicated that the low-intensity expression of ACP S-malonyltransferase *fabD* and the high-intensity expression of acetyl-CoA carboxylase and long-chain fatty acid-CoA ligase LcfA were more conducive to the efficient synthesis of iturin A. In future studies, several other strategies, such as strengthening precursor amino acid supplies, blocking by-product synthesis, and rewiring transcriptional factors, also need to be systematically optimized for efficient production of iturin A.

## Conclusion

Iturin A is a promising biosurfactant with various applications; however, low synthesis capability limits its production and application development. Fatty acids play a critical role in cellular metabolism and target product syntheses. In this study, we enhanced the expression of acetyl-CoA carboxylase, ACP S-malonyltransferase, soluble acyl-ACP thioesterase, and long-chain fatty acid-CoA ligase in fatty acid synthesis pathways, and the contents of fatty acids in the superimposed engineering strain HZ-ADFTL2 were increased significantly, which led to 6.59-fold increases of iturin A yield, reaching 2.96 g/L. Our results confirmed that strengthening fatty acid syntheses was an efficient approach for iturin A production, and this research provided a promising strain for industrial production of iturin A.

## Data Availability

The authors acknowledge that the data presented in this study must be deposited and made publicly available in an acceptable repository, prior to publication. Frontiers cannot accept a manuscript that does not adhere to our open data policies.

## Abbreviations

PYR, pyruvate; KIV, 2-keto-isovalerate; KMV, 2-keto-3-methylvalerate; OAA, oxaloacetate; α-KG, α-ketoglutaric acid; HPLC, high-performance liquid chromatography; SOE-PCR, splicing by overlap extension–polymerase chain reaction.
