# Supplementary material for: Enhanced production of iturin A by strengthening fatty acid synthesis modules in Bacillus amyloliquefaciens
Source: Front Bioeng Biotechnol. 2022 Sep 9;10:974460. doi: 10.3389/fbioe.2022.974460 (PMC9500472; doi:10.3389/fbioe.2022.974460)
Supplement: Supplementary file 1 [file Table1.DOCX]

**Enhanced production of Iturin A by strengthening fatty acid synthesis modules in *Bacillus amyloliquefaciens***

Lin Gao ^a, b^, Menglin She ^a^, Jiao Shi ^a^, Dongbo Cai ^a^, Dong Wang ^a^, Min Xiong ^a^, Guoming Shen ^b^, Jiaming Gao ^c^, Min Zhang ^d^, Zhifan Yang ^a*^, Shouwen Chen ^a, d*^

*^a^ State Key Laboratory of Biocatalysis and Enzyme Engineering*, *Environmental Microbial Technology Center of Hubei Province*, *College of Life Sciences*, *Hubei University*, *Wuhan, 430062*, *PR China*

*^b^ Tobacco Research Institute, Chinese Academy of Agricultural Sciences*, *Qingdao*, *266101*, *PR China*

*^c^ Hubei Corporation of China National Tobacco Corporation, Wuhan, 430000, PR China*

*^d^* *Key Laboratory of Green Chemical Technology of Fujian Province University*, *College of Ecological and Resource Engineering, Wuyi University, Wuyishan 354300, PR China*

*Corresponding author: Prof. Shouwen Chen and Prof. Zhifan Yang

Tel./fax.: +86 027-88666081.

*E-mail address*: mel212@126.com (S. Chen) and sailyangzhf@hubu.edu.cn (Z. Yang).

*Postal address*: 368 Youyi Avenue, Wuchang District, Wuhan 430062, Hubei, PR China

**Table S1 The primers used in this research**

| Primer name | Sequence 5′→3′ |
| --- | --- |
| pHY-F | GTTTATTATCCATACCCTTAC |
| pHY-R | CAGATTTCGTGATGCTTGTC |
| P43-F | TGATAGGTGGTATGTTTTCG |
| P43-R | TCATGTGTACATTCCTCTC |
| AccAD-F | GTGGCTGCAAGATTAGAATTT |
| AccAD-F | CTAATCTTGCAGCCACTCATG |
| AccBC-F | ATGTTAAAAATCAACGAAATTC |
| AccBC-F | TTATGAGCCCATTACATCATATG |
| TamyL-F | AAGAGCAGAGAGGACGGATT |
| TamyL-R | GCCGCAATAATGCCGTCGCACTG |
| AccAD-UF | CATTTGTTAAAGGATATATTCA |
| AccAD-UR | AGCGCAGAGCTTGTCTTTGCCAT |
| AccAD-DF | GTGGCTGCAAGATTAGAATTTG |
| AccAD-DR | GTTCTTGGCGATGGCTTCACTTTG |
| PbacA-F | CCTGCGATTTCGGCGAGATTCAA |
| PbacA-R | ATAAAAATTCTCCTTTTTGATAAAA |
| Pdual3-F | GAAATATTGATGTGACACTTGAA |
| Pdual3-R | TGATCCTTCCTCCTTTAGATCTG |
| FabD-F | ATGCTTACGTTTGTGTTCCCG |
| FabD-R | TCAGCCTGTAATCGATACCAGC |
| FabD-UF | CTTTTCGTTCTGTGACTTTTTT |
| FabD-UR | ATATACACTGCAATCATTTTTA |
| FabD-DF | ATGCTTACGTTTGTGTTCCCGG |
| FabD-DR | TCTCACGCCGGCTTTTTCAAAGGC |
| TesA_Ba_-F | ATGAAACAACCGCAATATATTGA |
| TesA_Ba_-R | ATGATGAACTTCAACAATGTTT |
| TesA_Ec_-F | TTATGAGTCATGATTTACTAAAG |
| TesA_Ec_-R | TCAGGCGAGCAGAATGCCTT |
| TesA_Cg_-F | ATGTCTGATAATCCGCATGAG |
| TesA_Cg_-R | CTAGTCGATCTGCAGTGGAACC |
| T2-TesA-F | TGATAGGTGGTATGTTTTCG |
| T2-TesA-R | GCCGCAATAATGCCGTCGCACTG |
| LcfA-F | ATGCAGTCTGAAAAGCCATGGC |
| LcfA-R | TTAAGGCACTTTGTTTTCAC |
| LcfA-UF | TCATCGGACTTATTTATCTGGTC |
| LcfA-UR | TGGAATACCGTCATGACCATTT |
| LcfA-DF | ATGCAGTCTGAAAAGCCATGGCTT |
| LcfA-DR | TTCGTGGCCGTCATAATCAATA |

**Fig. S1 The concentrations of total reducing sugars during iturin A fermentations.**

**
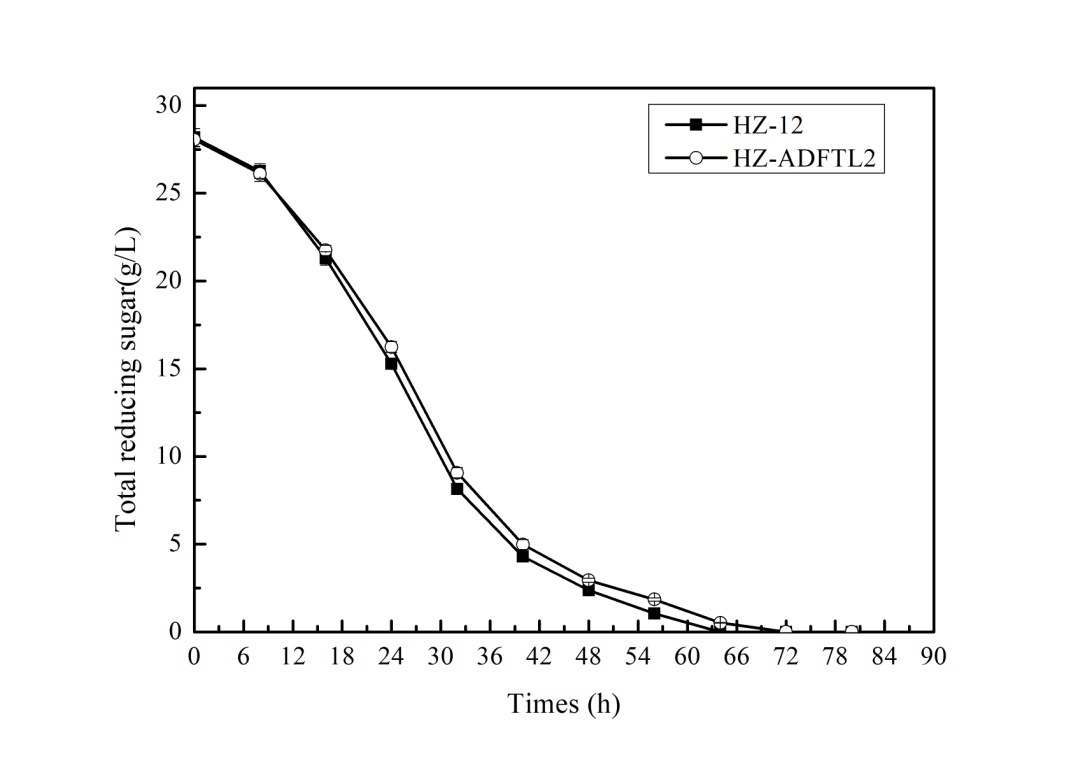
**

**Fig. S2 The iturin A yields produced by strains HZ-12 and HZ-ADFTL2 in ME medium.**

**
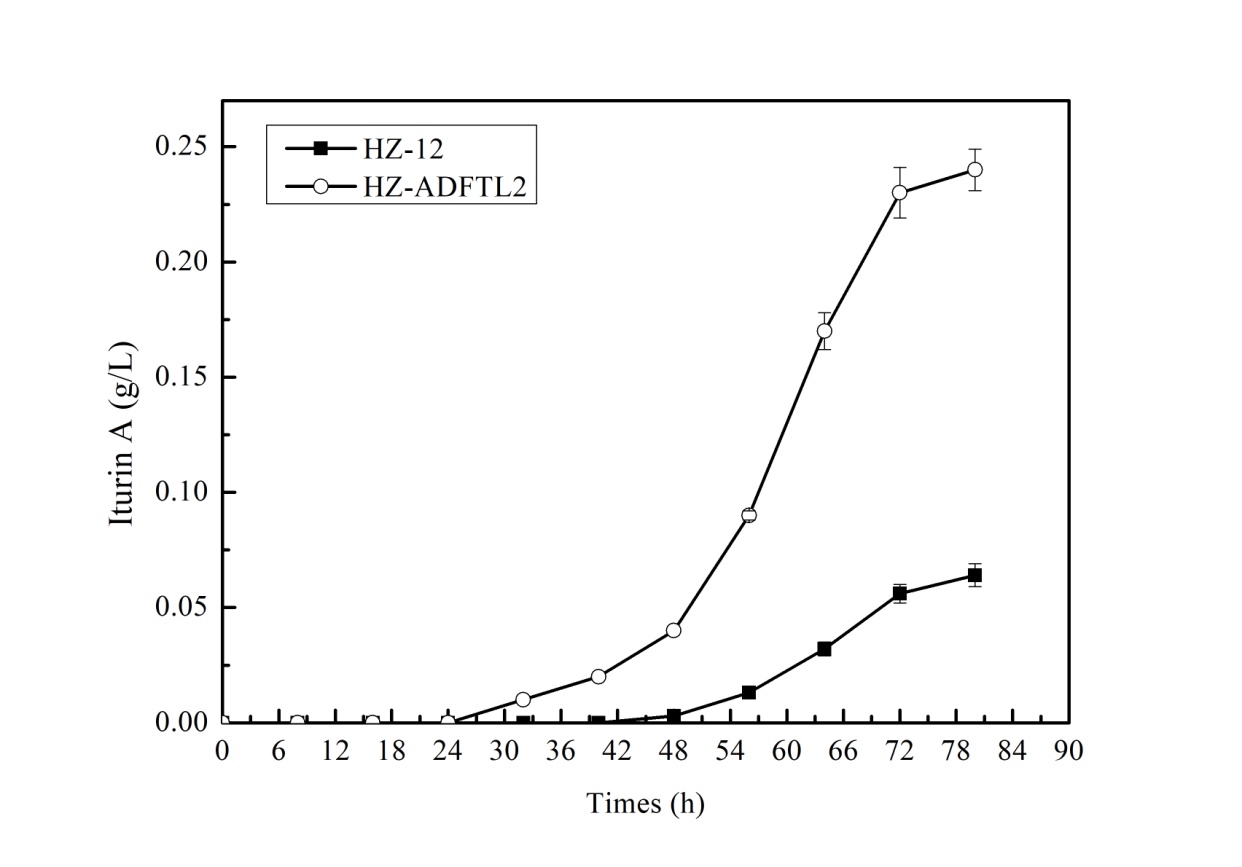
**

**Fig. S3 The concentrations of free fatty acids of strain HZ-ADFTL2.**

**
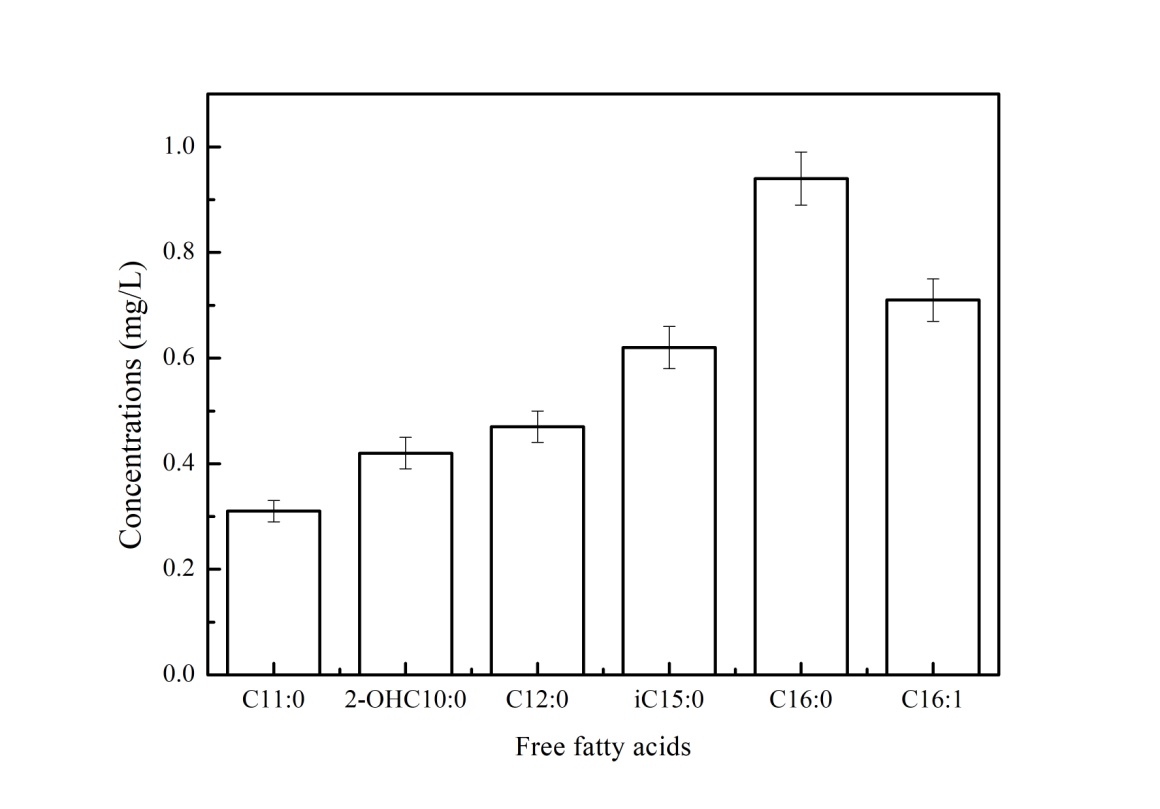
**
